# Changes and challenges in sexual life experienced by the husbands of women with breast cancer: a qualitative study

**DOI:** 10.1186/s12905-022-01906-8

**Published:** 2022-08-02

**Authors:** Maryam Maleki, Abbas Mardani, Mansour Ghafourifard, Mojtaba Vaismoradi

**Affiliations:** 1 Pediatric and Neonatal Intensive Care Nursing Education Department, School of Nursing and Midwifery, Tehran University of Medical Sciences, Tehran, Iran; 2 Department of Nursing, School of Nursing and Midwifery, Shahroud University of Medical Sciences, Shahroud University of Medical Sciences, Shahroud, Iran; 3 Nursing Care Research Center, Department of Medical Surgical Nursing, School of Nursing and Midwifery, University of Medical Sciences, Tehran, Iran; 4 Department of Medical‐Surgical Nursing, Faculty of Nursing and Midwifery, Tabriz University of Medical Sciences, Tabriz, Iran; 5 Faculty of Nursing and Health Sciences, Nord University, Bodø, Norway

**Keywords:** Breast cancer, Men, Marital life, Husband, Sexual dysfunction, Sexual life, Quality of care

## Abstract

**Background:**

Breast cancer (BC) in women can bring various problems to their marital and family life. Sexual life based on the experiences of the husbands of women diagnosed with BC has not been fully understood. Therefore, this research aimed to explore changes and challenges in sexual life experienced by the husbands of women diagnosed with BC.

**Methods:**

A qualitative research was carried out on 18 men whose wives had been diagnosed with BC at reproductive age. They were selected using purposeful sampling and were interviewed using in-depth semi-structured interviews. Collected data were analyzed using the conventional content analysis method.

**Results:**

‘Sexual life suspension’ was the main theme of this research. Also, ‘unfulfilled sexual expectations’, ‘perceived barriers to satisfy sexual expectations’, and ‘efforts to adapt to sexual problems’ were subthemes.

**Conclusions:**

The husbands of women with BC need support to improve their sexual and marital relationships. Education and counseling about sexual life during the treatment of BC should be incorporated into the healthcare program.

**Supplementary Information:**

The online version contains supplementary material available at 10.1186/s12905-022-01906-8.

## Background

Breast cancer (BC) accounts for 30% of new cancer diagnoses and is one of the most common types of cancers [1, 2]. According to the World Health Organization (WHO), BC is the second leading cause of death from cancer in women [1]. The prevalence of BC has been estimated to increase from two million patients in 2018 to more than three million patients in 2046, representing 46% increase [1].

BC also accounts for 76% of cancer cases among Iranian women and the total number of BC diagnosis is 42 000. Annually, more than 7 000 new cases of BC are diagnosed [3]. It has been estimated that the incidence of BC in Iranian women will be tripled annually until 2030 [4]. More than 40% of Iranian women with BC diagnosis are in the age range of 40–50 years indicating a lower age at BC diagnosis compared to women in other countries [5]. The relative 5-year survival rate of these women has improved over the past 3 decades due to advances in early detection through increased awareness and the widespread use of mammography [6]. According to global statistics, the relative 5-years survival rate of BC has reached 90% [1]. Studies show that the five-year survival rate in Iranian women with BC varies from 51% to 76.5% [7–10].

The recent high survival rate of BC has attracted the attention of health researchers to the quality of life (QoL) and sexual life of these women and their families [11, 12]. Sexual problems can be the result of any type of cancer, but it is more common in women with BC, because the breast is the symbol of femininity and plays an important role in sexual pleasure and arousal [13]. BC treatment has a wide range of physical and psychosocial consequences and leads to dysfunction and unpleasant changes in women’s sexual function [14, 15]. For example, mastectomy can change the individual’s perceptions of body image and reduce sexual attractiveness and femininity [16]. Chemotherapy causes hair and weight loss and induces premature menopause. Moreover, radiotherapy causes pain and dermatitis, which decrease women’s libido [17]. It is believed that deficiencies in sex hormones and changes in the body image alter the sexual function and cause arousal disorders, painful intercourse, and sexual dissatisfaction [18].

BC is a complex health problem and is difficult to cope with [19]. BC diagnosis in women can cause different health consequences for the whole family especially for their spouses [20]. BC can directly influence the quality of marital relationships and sexual function [18, 21]. Therefore, BC is considered a ‘disease of couples’ or ‘relational cancer’. It causes challenges including anxiety, depression, and sexual dysfunction in the marital relationship that are experienced not only by women but by also by their husbands [22, 23]. Sexual relationship is an important part of marital relationships [24] and reduces emotional stress during BC treatment. It can improve psychosocial reactions to BC cancer diagnosis [25].

It is believed that couples experience more issues in their sexual relationships after BC diagnosis compared to before it [21]. A study on 1011 patients showed that 70% of women with BC suffered from sexual dysfunction during the treatment process [26]. BC survivors often avoid having sexual relationships with their partners, but some others prefer having a close and intimate sexual relationship without vaginal intercourse [19]. Moreover, BC-related changes can increase the emotional distance between couples. Related psychological stress reduces marital satisfaction [27].

The husbands of women with BC also experience many problems in their sexual life [20] that can have negative consequences for their emotional, psychological, and physical wellbeing [28]. They have to deal with and adapt to life changes and provide support to their wives and children [20]. Issues in their sexual intimacy and inclination to talk about their feelings and concerns can ignite frustration, anxiety, and communication problems [20] [29]. Therefore, their marital relationship should be strengthened to prevent more damages to their sexual relationships [29].

### Background in Iran

Cultural and religious factors can influence the psychosexuality of women and their husbands [17]. In the Iranian culture, the desire for having sexual relationships by women through requesting or showing interest is considered inappropriate. Also, the husband's preferences and satisfaction with sexual relationships are considered more important than the wife's satisfaction [21]. Since couples usually do not talk to each other about sexual issues, they do not reach an agreement on how sexual issues should be resolved [30]. Couples also are ashamed of talking about sexual issues with their healthcare providers. It causes that sexual problems to remain unrecognized and unresolved [31]. Therefore, couples should be assessed by healthcare professionals with regard to their sexual problems and receive recommendations to meet their needs [32]. Nevertheless, the Iranian healthcare system has not reached the optimal performance to proactively assess sexual problems among BC patients and their husbands [33].

The role of the husbands of women with BC to cope with BC has been emphasized [34], given that they are in the best position to identify challenges in their sexual life. Accordingly, healthcare providers can devise strategies to improve the couples’ adaptation to sexual problems during the treatment process [31]. Therefore, this study aimed to explore changes and challenges in sexual life experienced by the husbands of women diagnosed with BC.

## Methods

### Design and participants

A qualitative research using conventional content analysis was used. Qualitative content analysis helps describe and interpret textual data based on the systematic process of data coding. In-depth descriptive and well‐organized summary of research findings requires conducting qualitative research instead of quantitative research [35]. The article was reported using the standards for reporting qualitative research (SRQR) guideline [36].

The participants were selected using purposive sampling from June 2019 to February 2020 in an urban area of Iran based on the following eligibility criteria: men living with their wives diagnosed with BC, being at stages 1–3 of BC, and undergoing BC treatment for the past 1–5 years. The presence of mental and other chronic diseases in men and their wives led to their exclusion.

### Ethics considerations

The ethical approval was obtained from the Ethics Committee affiliated with Shahroud University of Medical Sciences (decree number: IR.SHMU.REC.1398.012). Also, authorities granted permissions to enter the hospital before getting access to the patients’ medical files. Sufficient explanations were given to the participants about the research purpose, voluntary nature of participation in this study, anonymity and right to withdraw from the study at any time, and confidentiality of collected data. Written informed consent and permission to audio-record the interviews were obtained from the participants before data collection.

### Data collection

Two researchers (MM, AM) decently reviewed all medical files of the women diagnosed with BC who completed their treatments in the past five years in a referral hospital. Among 156 reviewed medical files, 52 of them had initial eligibility criteria. Next, the husbands of these women were contacted via phone call to review their additional eligible criteria. Those participants who met the full inclusion criteria were invited to be interviewed. The recruitment process was continued until data saturation was reached when data analysis did not lead to the exploration of new findings, which happened after 18 interview sessions.

In-depth semi-structured interviews using open-ended questions were carried out by the male researcher (AM) in Farsi. He scheduled appropriate time and place convenient to the participants to ensure of not interfering in their daily life routines. The interview guide (Additional file 1) consisted of questions that helped with collecting in-depth data about the research phenomenon. The main focus of the questions was changes happened in the life and sexual relationships after BC diagnosis. These questions were compiled by the research team after conducting a literature review on sexual life and its related issues among BC survivors. The depth of interviews was improving through asking probing questions to follow up the participants’ perspectives.

The interviews lasted 45–75 min and 14 participants were interviewed once. Four other participants were interviewed twice to remove ambiguities in the data collection process. Therefore, 22 interviews were performed with 18 participants. All interviews were recorded using a digital audio recorder.

Questions about the participants’ sociodemographic characteristics including the participant’s age, education level, duration of the marriage, number of children, place of residence, employment status, economic status, the participants’ wife’s age, time passed from BC diagnosis, and cancer treatment modalities were asked before the interviews.

The researchers were faculty members of nursing and midwifery schools at the time of the study. They were experts in qualitative research and had previous experiences with conducting qualitative research in cancer care. Two researchers (MM, AM) have clinical work experiences with cancer patients, but they had no relationship with the study participants before the research. They wrote reflective notes to bracket their own assumptions regarding the study phenomenon.

### Data analysis

Verbatim transcribing of the interviews was performed immediately by the responsible researcher (AM) and simultaneously was entered into data analysis by the research team (MM, AM, MG, MV) applying conventional qualitative content analysis [37, 38]. To immerse in the data and gain an in-depth understanding of the interviews’ content, the transcripts were read line-by-line several times. The meaning units were derived from the transcriptions and were labeled through open coding. Codes were assigned into categories based on their similarities. The codes and categories were compared together and concerning the whole data using the constant comparison method to develop the main theme and related subthemes [35, 38].

### Trustworthiness of data

The four components of trustworthiness for qualitative research suggested by Lincoln and Guba including credibility, transferability, dependability, and confirmability were applied. Strategies used to strengthen the credibility of this study were prolonged engagement with the research settings and participants, reflexivity, peer debriefing, and member checking. Also, the interviewer wrote reflective notes to bracket his own assumptions about the study phenomenon and ensure that the research findings reflected the participants’ perspectives. For peer debriefing, a third researcher reviewed and assessed the data analysis process. Also, a summary report of research results was provided to two participants who confirmed that our findings demonstrated their perspectives. Transferability was ensured through the provision of a thick description of the research findings. For dependability and confirmability, audit trial was used. An impartial person who was expert in qualitative research was asked to review and assess the transcripts, data analyses, and findings [39].

## Results

The participants were married and had the age range of 42–57 years (50.33 ± 4.15 y). The mean duration of their marriage was 21.16 years (SD = 5.44 y). Most of them had one child or 2 children (66.6%), resided in the city (72.2%), and had an under-diploma education degree (50%). Their wives had an age range of 33–50 years (44.38 ± 4.32 y). BC diagnosis happened in 56.6% and 44.4% of the participants’ wives more than 3 years ago and in the last 1–3 years, respectively. The majority of the participants’ wives (88.9%) had undergone mastectomy (Table 1).

**Table 1:** The demographic characteristics of the participants

Variable	Value
Age, range (year)	42–57
Age (y), mean (SD)	50.33 (4.15)
Duration of marriage (y), mean (SD)	21.16 (5.44)
*Education level*	N (%)
Under diploma	9 (50)
Diploma	3 (16.7)
Academic	6 (33.3)
*Place of residence*	
City	13 (72.2)
Village	5 (27.8)
*Number of children*	
No children	2 (11.1)
One child or 2 children	12 (66.6)
Three or more children	4 (22.3)
*Occupation*	
Employed	8 (44.4)
Retired	8 (44.4)
Unemployed	2 (11.1)
*Economic status (self-report)*	
Sufficient	5 (27.8)
Relatively sufficient	10 (55.6)
Not sufficient	3 (16.6)
*Participants’ wives*	
Age, range (y)	33–50
Age (y), mean (SD)	44.38 (4.32)
*Time passed from cancer diagnosis*	N (%)
1–3 years	8 (44.4)
More than 3 years	10 (55.6)
*Mastectomy*	
Yes	16 (88.9)
NO	2 (11.1)
Chemotherapy	18 (100)
*Radiotherapy*	
Yes	10 (55.6)
No	8 (44.4)

**Fig. 1:**
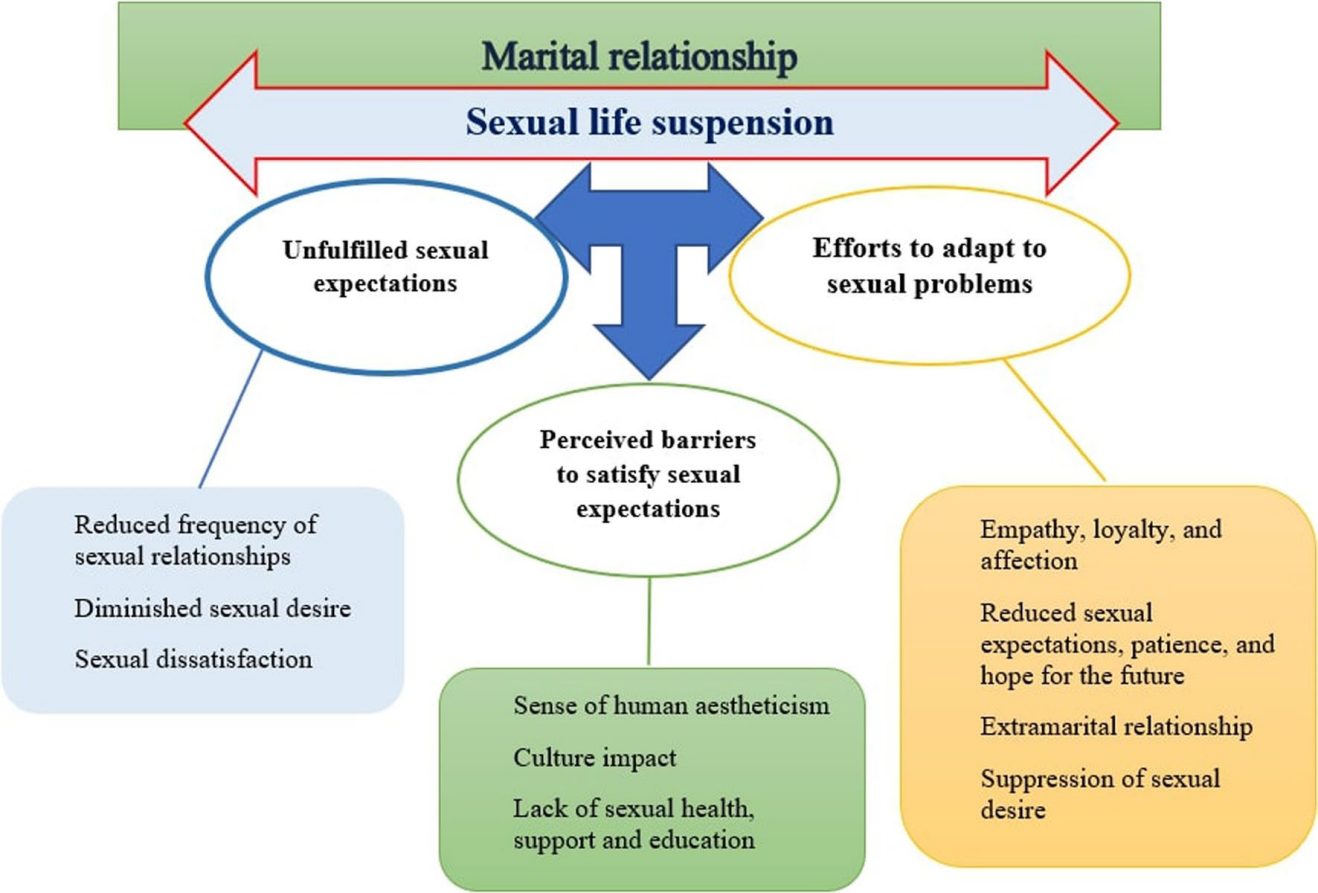
Themes, subthemes and key codes developed in this research

The participants experienced substantial changes in their sexual life due to the diagnosis and treatment of BC in their wives. Our research findings consisted of the main theme of ‘sexual life suspension’ and three subthemes of ‘unfulfilled sexual expectations’, ‘perceived barriers to satisfy sexual expectations’, and ‘efforts to adapt to sexual problems’ (Fig. 1, Table 2).

**Table 2:** A summary of themes, subthemes and key codes

Theme	Subthemes	Key codes
Sexual life suspension	Unfulfilled sexual expectations	Reduced frequency of sexual relationships Diminished sexual desire Sexual dissatisfaction
	Perceived barriers to satisfy sexual expectations	Sense of human aestheticism Culture impact Lack of sexual health, support and education
	Efforts to adapt to sexual problems	Empathy, loyalty, and affection Reduced sexual expectations, patience, and hope for the future Extramarital relationship Suppression of sexual desire

### Sexual life suspension

BC severely affected the sexual life of the men following the occurrence of changes in the well-being and sexual health of their wives. After the diagnosis of BC, major changes occurred in the couples’ marital life leading to various challenges. There were barriers to meet their sexual needs, but each person responded differently to them. Therefore, the sexual life of the men was suspended.

### Unfulfilled sexual expectations

The participants mostly reported a normal sexual life before the onset of BC. Some other men complained about sexual coldness in their wives even before BC.

“Before this disease, my wife and I had sexual relationships almost every 10 or 15 days and we experienced no problem in our married life.” (Participant (P) 6, 48 years old)

“We did not have an understanding about sexual issues at the beginning of BC. My wife was sexually very cold, and she did not like to have any sexual relationship. We had a sexual relationship once or twice a month, it reduced greatly after BC diagnosis.” (P 8, 49 years old)

Negative changes in their sexual relationships following BC diagnosis were characterized as a reduction in sexual desire, frequency of sexual relationships, and sexual dissatisfaction.

A sharp reduction in the frequency of sexual relationships was reported indicating having no sexual relationships during a year. It was attributed to the consequences of BC treatment including decreased physical charm and libido, vaginal dryness, and painful intercourse.

“Before my wife's disease, we had sex every 7-10 days, but my wife felt often bored, was not interested in having sex, was harassed during intercourse; then the frequency of our sexual relationship decreased a lot ... and this is now once or twice a year.” (P 10, 45 years old)

“The frequency and duration of our sexual intercourse decreased a lot after my BC diagnosis. She [wife] had no longer the beauty and freshness. I think if my wife had not been ill, the quality of our sexual intercourse would become much better.” (P 15, 51 years old)

BC had negative consequences for their wives’ sexual desire. The men wanted to have sex and satisfy their own sexual needs, but their sexual desire for their partner had been diminished, though their instinctive sexual desire was high.

“Before the disease, we had sex twice a week, even three times a week, but at this moment we do not have sex ... my sexual desire is fine ... no ... but I do not want to have sex with my wife anymore.” (P 11, 48 years old)

The occurrence of BC was a great shock to the participants’ life and affected all aspects of their life so that their sexual desire toward their wives decreased. Therefore, their wives more often asked for having sexual relationships.

“The diagnosis of BC was a great shock to us. It had a great impact on my sexual desire. When one breast is not in place and you can see the wound, an unpleasant feeling comes to you ... I always overcame my wife sexually ... I had a lot of sexual desire for my wife, but I do not have much desire now unless it takes three weeks or a month for me to request a sexual relationship.” (P 9, 51 years old)

Given the reduction in the participants’ sexual desire for their wives and the frequency of sexual relationships, their sexual needs were not met leading to sexual dissatisfaction.

“My sexual expectations in the marital relationship have not been really met after BC diagnosis. Breast removal and many other things related to my wife's illness suppressed my sexual desire. Naturally, I withdrew myself, so I have no longer sexual satisfaction.” (P 2, 52 years old)

### Perceived barriers to satisfy sexual expectations

Barriers to meet the participants’ sexual needs were the sense of human aestheticism, culture, and insufficient sexual health support and education by healthcare providers.

Changes in their wives’ appearance were the basis of a series of new changes in their sexual life. Their wives had no longer those previous beauties and charm. On the other hand, the participants' innate sense of aestheticism, like any human being who was attracted to beautiful phenomena, inevitably reduced the sexual attraction of men to their wives. The presence of this innate sense was an important obstacle to have sexual relationships.

“Appearance changes such as hair loss affected my sexual relationships. Suppose you had a beautiful woman ... now she has no hair ... her breasts are removed ... well, you do not like to touch her ... If I go to my wife, she will be aroused ... but I do not go ... I no longer have that state of lust.” (P 1, 54 years old)

“I love beauty and my wife has no longer that beauty … a woman who until 6 months ago, for example ... combed her hair ... arranged her eyebrows ... did makeup … but now my wife has no beauty to persuade me to go to her.” (P 18, 43 years old)

Culture as an integral part of human life influenced sexual life. Concubine as to have extramarital sexual relationships was culturally unacceptable in the Iranian society. Therefore, it hindered finding a sexual partner at a time when the men were unable to meet their sexual needs with their wives.

“Having sex with another woman is culturally unacceptable here. Others may say look at this man, his wife is still alive, but she is just sick and he has sexual relationships with another woman. In my opinion, this is not a problem at all, but the society does not accept it.” (P 17, 57 years old)

“Our culture is very influential in sexual matters. I cannot think of another woman when I have a wife, even if having such a relationship is not a problem in terms of religion. Where I really cannot have a sexual relationship with my wife, my hands are tied up. I have to endure this pressure.” (P 6, 48 years old)

“I dare not to have sex with another woman, because the society does not accept it. My reputation will be ruined and people may say that my pants have been doubled [proverb: it means he has two wives]!” (P 9, 51 years old)

The participants needed to receive support, training, and information on sexual issues from healthcare providers. They acknowledged that they had not received any support or training about it.

“Healthcare staff was supposed to talk to us in the hospital about sexual problems, but later they said ‘no consultation about it could be provided’. No one talked to us about it anymore. I did not know where to go and what to do.” (P 12, 55 years old)

“They [healthcare providers] did not inform us about the sexual relationship. The doctor only said ‘you should not have children’ and ‘getting pregnant means death.’” (P 4, 48 years old)

Some participants turned to unofficial information sources after that they did not find answers to their inquiries about sexual problems.

“When I found out that my wife and I had no problem in sexual intercourse, but my wife vagina was dry, I searched on the internet and found out a series of gels that could alleviate vaginal dryness.” (P 3, 52 years old)

### Efforts to adapt to sexual problems

The participants experienced sexual crisis and used a variety of adaptation measures to overcome it. For instance, they imagined their wives’ condition and made empathy.

“I thought to myself what my wife would have done if this had happened to me! I expected that my wife should deal with it and should not leave me alone. Therefore, I decided to cope with it.” (P 14, 50 years old)

Loyalty to and affection for their wives facilitated coping with sexual problems.

“I have a belief that if someone gets married to someone, he must remain faithful to her and stay by her side. If my wife does not even have two hands, I deal with it. It is so unscrupulous and cowardly that I leave my wife, because my sexual needs are not met.” (P 11, 48 years old)

“I do allow myself to look at her breasts so that she does not feel embarrassed. I act as if nothing has happened at all and it does not matter to me. I am ready to sell my eye cornea for her to get recovered. I love her so much that I have dedicated my life to her.” (P 12, 55 years old)

To deal with the sexual crisis, the participants reduced their sexual expectations from their wives. They dreamed a day when everything would return to normal.

“After my wife's illness, our marital and sexual life was completely destroyed. The situation became very difficult for me and I hoped that the situation would change over time. I am just waiting for that our sexual relationship gets improved.” (P 4, 48 years old)

“I should reduce my sexual expectations. There is no other way and I think this is the best strategy.” (P 10, 45 years old)

Although the participants were committed to have sexual relationships only with their wives, some of them had sexual relationships with another sexual partner in the face of sexual crisis and to meet their sexual needs.

“During my wife's treatment, when we could not have sex for a long time, I went looking for another partner. This was only to meet my own sexual needs.” (P 7, 42 years old)

One of the most difficult adapting behaviors was the suppression of sexual desire. The participants experienced sexual helplessness as they had no choice, but to suppress their sexual desire.

“I have suppressed my sexual desire for a long time, because of my wife's illness, and I try not to think about it anymore.” (P 16, 54 years old)

“My sexual need is not met. I cannot do anything special, but to suppress it.” (P 13, 55 years old)

## Discussion

A few qualitative studies so far have addressed sexuality and sexual health among men after the diagnosis of BC in their wives. This qualitative research explored changes and challenges in sexual life experienced by this vulnerable group. We found that men experienced unfulfilled sexual expectations given the occurrence of significant changes in their sexual relationships. Barriers to meet their sexual needs and efforts made by them to adapt to sexual changes were explored.

Unfulfilled sexual expectations consisted of a reduction in the frequency of sexual relationships, diminished sexual desire, and sexual dissatisfaction. Similarly, a quantitative descriptive study on sexual adjustment among Israeli men after BC diagnosis showed that over 70% of them had difficulties in their sexual activities [40]. A qualitative study in the USA reported that men’s sexual desire for their wives diminished after BC diagnosis [41]. Furthermore, in another qualitative study in the Iranian context, men’s sexual desire decreased mostly due to mastectomy and treatment complications such as alopecia [31]. A cohort study revealed that the partners of young BC survivors had more sexual difficulties and less sexual enjoyment compared to the partners of healthy controls [42]. Undesirable sexual functions have been reported in studies on the sexual issues of women with BC. The majority of Chinese women with BC suffered from a significant reduction of sexual desire and frequency of sexual activities [43]. Changes in sexual life including reduced sexual relationships and sexual desire in BC survivors have been reported by many studies [44–47].

Barriers to meet sexual needs were described by our research participants. The participants' innate sense of aestheticism reduced their sexual attraction to their wives given the occurrence of changes in the women’s physical attractiveness. According to Nasiri et al.’s study, unpleasant senses experienced by men because of physical changes in their wives led to avoid close contact with their wives and having sexual relationships [31]. Men often get separated from their wives, because of the disease’s impact on their sexual relationships [14, 20]. Indian women with BC undergoing mastectomy also stated that their husbands had arousal difficulties [48]. BC treatments usually cause most women to feel sexually inadequate and incomplete, which is likely associated with marital separation and breakdown of sexual relationships [49].

Physical and appearance problems in women following the treatment of BC are the common sources of sexual problems in men [18, 19, 47]. Sexual attractiveness and body beauty for women are emphasized in some cultures. Brazilian men consider the body beauty of women as an ideal not only for marriage but also for sexual relationships [50]. Therefore, breast-conserving surgery has become a routine approach to BC treatment in recent years. However, some patients still require mastectomy to decrease the risk of BC relapse [51, 52]. A prospective controlled study suggested that those women who underwent a mastectomy were probably more at the risk of post-operative sexual dysfunctions [53]. Zehra et al. in a systematic review and meta-analysis reported that patients with breast-conserving surgery exhibited a better body image and physical and sexual health than those with mastectomy [54]. Therefore, it is important to pay attention to the quality of the surgery type because poor surgery-related cosmetic outcomes can impair sexual health [55].

In some cultures such as Brazil, having extra-marital sexual relationships in situations that wives are unable to meet their sexual needs has been suggested [44, 50]. However, having an extramarital sexual relationship is unacceptable in Islam and the Iranian culture [19]. The tradition of temporary marriage in Islam supports men’s decision to marry more than one woman. On the other hand, remarriage for men when their wives become ill is unacceptable in the Iranian culture and is dependent on fulfilling legal requirements [56]. Iranian women also forcefully disagree with their husband’s remarriage.

Lack of support, education, and training about sexual issues by healthcare providers was another perceived barrier in our study. Provision of adequate support and information concerning intimacy and sexuality can decrease distress in women with BC and their husbands [57]. This is a common finding in other studies that healthcare providers typically do not provide support and education about sexuality to patients with BC and their partners [49, 58–60]. There is a need to create an open, truthful, accepting communication environment with BC women and their husbands within the healthcare system and help them meet their sexual health needs [61, 62]. Education programs for healthcare providers can improve their knowledge of sexual issues related to BC and how to communicate them to patients and their husbands [61]. When sexual issues are discussed with healthcare providers, husbands may not always be present to hear. Written information regarding sexual issues can improve their knowledge of how to resolve sexual issues during BC treatment [59].

The participants experienced sexual crisis after the diagnosis of BC in their wives. Some of them used the strategies of empathy, loyalty, patience, and hope to normal conditions in the future. Some others went looking for sexual relationships with another sexual partner or suppressed their sexual desire in dealing with this crisis. The adopted strategies can be various based on religious beliefs and relationship contexts [47]. Muslims believe that illness is a kind of test of God and God wants to see if they can endure difficulties or deviate. A qualitative study on Iranian men after BC diagnosis in their wives reported that the suppression of sexual desire, toleration of sexual frustration, and loyalty to their wives helped overcome unmet sexual needs [31]. However, in the Taiwan context, women with BC reported that their husbands had illegal sexual affairs with another partner [45]. In addition, Malay women with BC proposed their husbands to marry another woman in order to meet their sexual needs [47]. Jones et al.’s study on Canadian women with BC showed the necessity of having appropriate empathy, and a greater understanding and awareness of their husbands throughout cancer trajectory [63].

## Limitations

The present study is the pioneer of exploring men's sexual changes and challenges after BC diagnosis in their wives. Some limitations might have affected our data collection and analysis. Our participants’ experiences might not be the representative of men with wives diagnosed with BC that would be outside of the reproductive age. The researchers bracketed their presumptions and ideas using reflexivity, but the researchers’ subjectivity inevitably might have affected the interview process. This condition is especially relevant in the current study since the interviewer has been a qualified nurse and had a specific interest in this topic. The participants were recruited from a hospital in an urban area of Iran, which could impact on the transferability of our findings to other contexts. Taboo attached to sexual and marital issues in the Iranian culture might have caused the concern of disconnection of the alliance between the researcher and the participants and could hinder collecting in-depth data about the research phenomenon.

## Conclusion

This study improves our knowledge of sexual changes and challenges experienced by the husbands of women diagnosed with BC. Following the diagnosis of BC, major changes and challenges occur in the marital life of women with BC and their husbands, which suspend men’s sexual life. Our findings inform healthcare providers about the significance of paying attention to sexual health problems experienced by men during BC treatment. They should provide opportunities for the husbands of women with BC to express their concerns about their sexual health issues. This will promote help-seeking behaviors in this vulnerable group.

It is also recommended that topics concerning the sexual life of women with BC and their husbands are incorporated into healthcare programs aiming at the improvement of QoL in couples. Education and counseling about sexual relationships during the treatment of BC should be one part of the holistic program aiming at the improvement of couple’s sexual life and should be easily accessed by them in community settings. Supportive interventions by healthcare professionals for the husbands of woman with BC hinder further damages to marital relationships between couples.

Future research is needed to design strategies for the provision of appropriate support to women with BC and their husbands, and examine their effects on couples’ sexual life. In addition, future research should be conducted in other contexts. They can consider the development of appropriate practical instruments for the investigation of sexual life among the husbands of women with BC.

## Supplementary Information

**Additional file 1**. Interview guide.

## Data Availability

The datasets used and/or analysed during the current study available from the corresponding author on reasonable request.

## Abbreviations

BC: Breast cancer
P: Participant
QoL: Quality of life
SRQR: Standards for reporting qualitative research
WHO: World Health Organization
