# Supplementary material for: Changes and challenges in sexual life experienced by the husbands of women with breast cancer: a qualitative study
Source: BMC Womens Health. 2022 Aug 2;22:326. doi: 10.1186/s12905-022-01906-8 (PMC9344622; doi:10.1186/s12905-022-01906-8)
Supplement: Supplementary file 1 — Additional file 1. Interview guide. [file 12905_2022_1906_MOESM1_ESM.docx]

**Interview guide**

*Interview questions*

- What changes happened in your sexual relationships after that your wife was diagnosed with BC?
- What changes have happened in your attitude toward sexuality after the diagnosis of BC?
- What issues have you experienced in terms of sex and sexual behaviors after BC diagnosis?
- Has your wife been able to meet your sexual expectations and vice versa after BC diagnosis?

*Probing questions*

- Can you give me an example?
- Can you explain it more?
